# An Eschar-like souvenir from a journey to Colombia: Ecthyma gangrenosum as a differential diagnosis of tropical diseases in immunocompromised patients – a case report

**DOI:** 10.1186/s12879-021-05998-9

**Published:** 2021-04-12

**Authors:** Gabriela M. Wiedemann, Jochen Schneider, Mareike Verbeek, Björn Konukiewitz, Christoph D. Spinner, Henrik Einwächter, Roland M. Schmid, Kathrin Rothe

**Affiliations:** 1 Department of Internal Medicine II, University hospital rechts der Isar, Technical University of Munich, School of Medicine, Munich, Germany; 2 German Center for Infection Research (DZIF), partner site Munich, Munich, Germany; 3 Department of Internal Medicine III, University hospital rechts der Isar, Technical University of Munich, School of Medicine, Munich, Germany; 4 Institute of Pathology, Technical University of Munich, School of Medicine, Munich, Germany; 5 Institute for Medical Microbiology, Immunology and Hygiene, Technical University of Munich, School of Medicine, Munich, Germany

**Keywords:** Ecthyma gangrenosum, *Pseudomonas aeruginosa*, Tropical disease, Skin ulcer, Leukemia, Ibrutinib, Case report

## Abstract

**Background:**

Ecthyma gangrenosum (EG) is a cutaneous infectious disease characterized by eschar-like skin ulcers typically caused by *Pseudomonas aeruginosa*. Here, we report a case of relapsing EG in a patient who had returned from a trip to Colombia, thus establishing EG as an important differential diagnosis of tropical diseases, and demonstrating that even long-term antibiotic treatment can result in only partial remission of EG.

**Case presentation:**

A 77-year-old man with underlying chronic lymphocytic leukemia (CLL) on ibrutinib treatment was admitted because of a superinfected mosquito bite on the left ear and multiple partially necrotic skin lesions disseminated all over the entire body five days after returning from a trip to Colombia. The initial clinical suspicion of a tropical disease (leishmaniosis, systemic mycosis, or others) could not be confirmed. During the diagnostic workup, microbiological cultures of the skin biopsies and bronchoalveolar lavage revealed *Pseudomonas aeruginosa*, leading to a diagnosis of EG. Initial antibiotic treatment resulted in partial remission. However, the patient had to be re-admitted due to a relapse 3–4 weeks after the first episode. Finally, the patient was successfully treated with a combined approach consisting of antibiotics, recurrent surgical incisions, and administration of immunoglobulins.

**Conclusions:**

In conclusion, EG should be considered as a differential diagnosis in immunosuppressed patients presenting with eschar-like skin ulcers. A combined treatment approach seems to be the best choice to achieve clinical cure and avoid relapse.

## Background

Ecthyma gangrenosum (EG) is a rare infectious disease of the skin mainly caused by *Pseudomonas aeruginosa (P. aeruginosa)* [1]. In the majority of the cases, EG develops in immunocompromised patients, such as patients with hematologic neoplasia, immunodeficiency syndromes, and patients receiving chemotherapy or immunosuppressive therapy [1–4]. Clinical manifestations of EG characteristically begin with erythematous skin lesions, which subsequently proceed to hemorrhagic bullae with necrotic areas or central black eschars, often surrounded by an erythematous halo [1–3, 5, 6]. Cultures of the skin lesions typically reveal *P. aeruginosa,* although co-detection of other bacteria or fungi is possible, and it is acknowledged that *P. aeruginosa* is not the only etiological agent of EG [1, 7]. Treatment of EG consists of antimicrobials and surgical debridement. The treatment must be started in a timely manner upon diagnosis and adjusted according to the antibiotic susceptibility tests of the etiological pathogens, as accompanying bacteremia is associated with poor prognosis [1, 2]. On this basis, we present a case of relapsing EG in a non-neutropenic patient with chronic lymphocytic leukemia (CLL).

## Case presentation

**Fig. 1:**
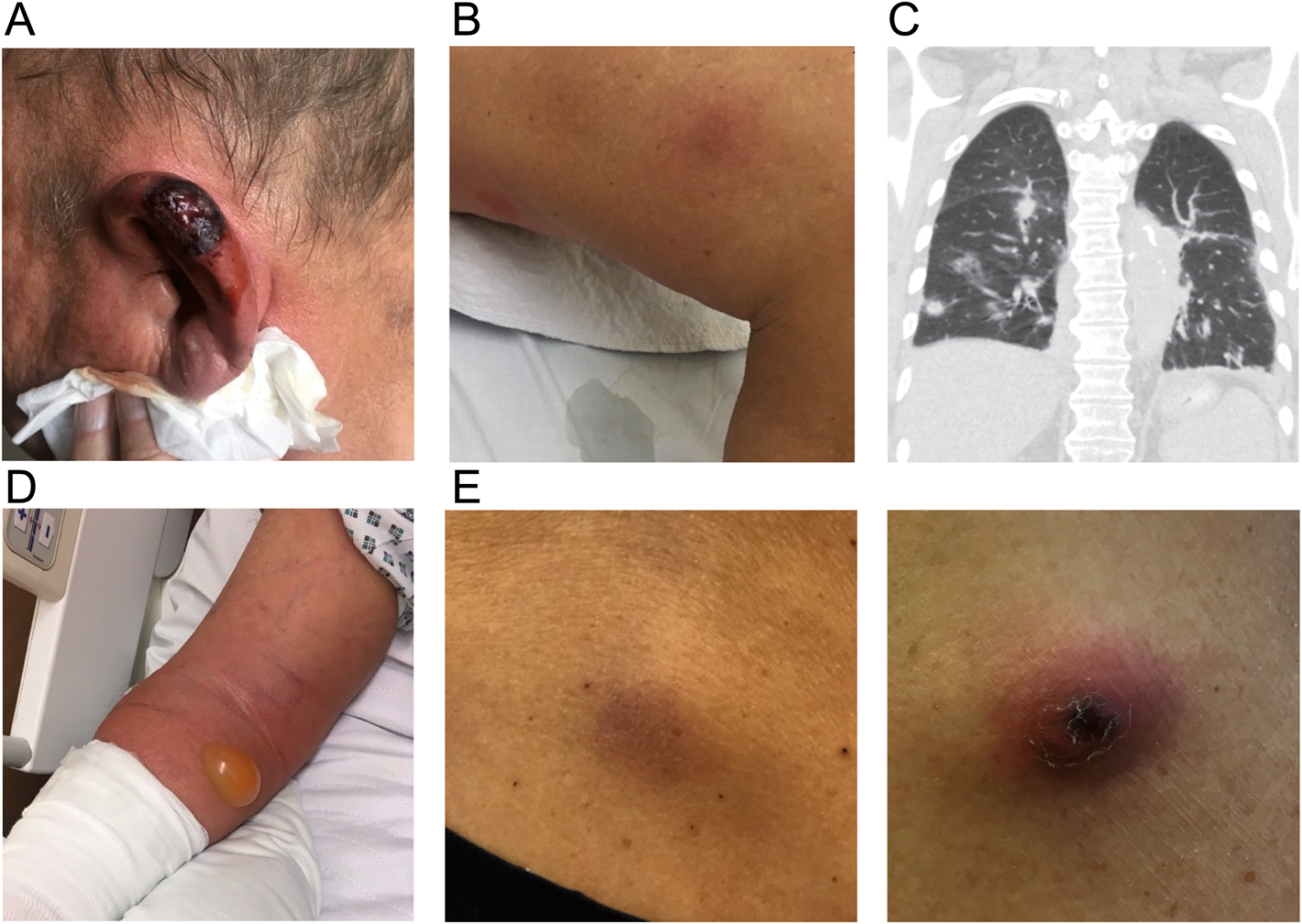
Skin and lung lesions caused by *P. aeruginosa*. **a** Livid discoloration of the left ear on day 0. **b** Erythematous skin lesions on the patient’s leg. **c** Chest CT scan showing bilateral pulmonary lesions. **d** Phlegmonous inflammation of the right arm. **e** Skin lesions upon secondary presentation before surgical incision

**Fig. 2:**
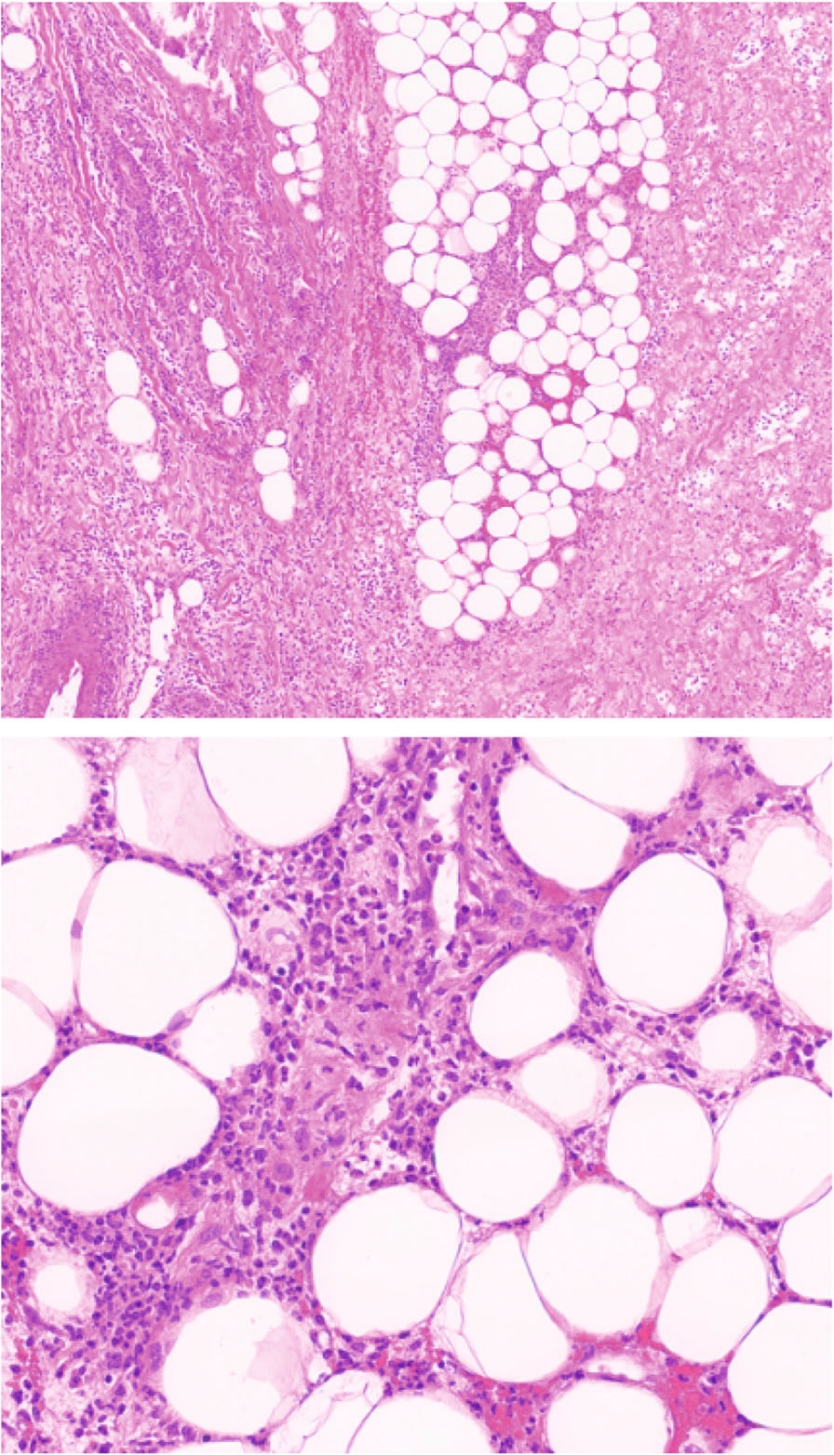
Hematoxylin and eosin (H&E) stained tissue section of the right forearm showing acute phlegmonous inflammation

**Fig. 3:**
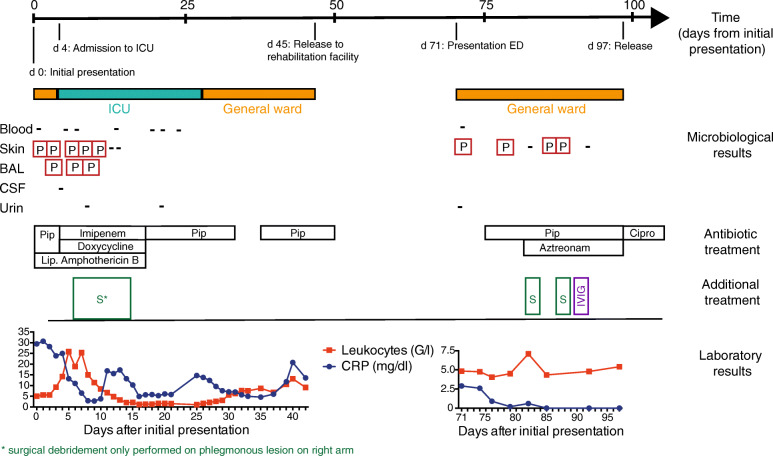
Schematic showing the treatments, microbiological, and laboratory findings over the course of both hospital admissions. The location “skin” includes wound swabs, punctates, and biopsy specimen. Abbreviations: ED: emergency department, CPR: cardiopulmonary resuscitation; CSF: cerebrospinal fluid; P: *Pseudomonas aeruginosa*; Ef:*Enterococcus faecium*; −: no bacterial growth; BAL: bronchoalveolar lavage; Pip: piperacillin; Cipro: ciprofloxacin; L: linezolid; Vanco: vancomycin; S: surgical incisions/ debridement; IVIG: intravenous immunoglobulin. Conversion in SI units: 1 mg/dL CRP = 95.2 mmol/L; 1 mg/dL creatinine = 88.4 μmol/L

A 77-year-old Caucasian man presented with a livid discoloration of the left ear, which had first appeared about five days prior to the patient’s flight home from Colombia, where he had spent a two-week vacation, including a stay of several days on a banana plantation in the department of Cundinamarca. The patient remembered a mosquito bite to his left ear during his stay in Colombia. Since his return, he had been experiencing increasing weakness and shortness of breath. The patient had been diagnosed with CLL four years earlier and was currently on ibrutinib therapy. Upon presentation, the patient was in a stable cardiorespiratory state (blood pressure 139/75 mmHg [18.5/10.0 kPa]), heart rate was 92 beats per minute, body temperature was 36.6 °C (309.8 K), and peripheral oxygen saturation (SpO_2_) was 100% with 2 L of oxygen. Physical examination showed a lividly discolored area on the left ear (Fig. 1a) and several erythematous, elevated, nodule-like skin lesions disseminated on the torso and extremities (Fig. 1b, including an eschar-like necrotic skin lesion on the right forearm. CLL treatment with ibrutinib was discontinued. Laboratory tests demonstrated abnormal serum electrolytes (sodium 132 mmol/L, potassium 3.2 mmol/L), acute renal insufficiency (serum creatinine 2.6 mg/dL [229.8 μmol/L], blood urea nitrogen 37 mg/dL [6.2 mmol/L]), mild anemia (hemoglobin, 13.2 g/dL [8.20 mmol/L]), metabolic acidosis (pH 7.353, HCO_3_ 17.6 mmol/L, lactate 3.9 mmol/L), and elevated C-reactive protein (CRP, 29.5 mg/dL [2809.5 mmol/L]). The leukocyte count was within the normal range with a relative increase in monocytes (leukocytes 5.04 G/L with 35% neutrophils, 31% lymphocytes, and 25% monocytes). A chest computed tomography (CT) scan revealed bilateral pulmonary lesions (Fig. 1**c**). Given the patient’s recent travel history and the eschar-like presentation of some of the lesions, a tropical disease was suspected. Skin biopsies were obtained from the right ear and right forearm after starting antimicrobial treatment with piperacillin/tazobactam (4.5 g tid) in combination with liposomal amphotericin B (400 mg/day) and doxycycline (100 mg bid) to provide empiric coverage against leishmania, endemic mycosis such as histoplasmosis, and atypical bacterial infections such as rickettsiosis. Within 24 h of the biopsy, the patient’s condition rapidly deteriorated. He developed further skin lesions as well as a rapidly progressive phlegmon starting from the biopsy site on the right forearm. The patient was transferred to the intensive care unit (ICU) due to septic shock and required high-dose catecholamine therapy (maximum dosis during shock: 600 μg/h norepinephrine and 1400 μg/h epinephrine) along with mechanical ventilation. Antibiotic therapy was escalated to imipenem, doxycycline, and amphotericin B. Surgical excision was performed on the progressively inflamed area on the right arm (Fig. 1**d**). Histological evaluation showed an acute phlegmonous infection with necrosis (Fig. 2). Blood cultures remained sterile. The skin specimens were tested for chagas, babesia, leishmania, endemic mycosis, tuberculosis, cryptococcosis, and rickettsia, but yielded negative results. Finally, microbiological cultures of the necrotic area on the patient’s ear, right forearm, and bronchoalveolar lavage (BAL) revealed the growth of *P. aeruginosa*, with comparable antibiotic susceptibility testing results showing susceptibility to piperacillin, ceftazidime, cefepime, carbapenems, aztreonam, and aminoglycosides. Thus, a diagnosis of Ecthyma gangrenosum was established. After 8 days of antibiotic treatment and almost daily surgical debridement of the right arm, the patient’s clinical condition significantly improved while the microbiological cultures remained sterile. Liposomal amphotericin B and doxycycline were stopped, and treatment with piperacillin (4 g tid) was continued. Surgical debridement was not performed for skin lesions apart from those on the right arm because these nodules either disappeared or significantly decreased in size, and the systemic inflammatory markers (CRP) also normalized with antimicrobial treatment. Finally, antimicrobial treatment was discontinued 50 days after the patient’s initial presentation, and he was transferred to a rehabilitation facility (Fig. 3).

Three and a half weeks after discontinuation of the antibiotic therapy, the patient presented again to the emergency department (ED) with an elevated CRP and recurrence of the skin lesions on the extremities and torso. Physical examination showed red bullous skin lesions with a liquid interior, some of which again displayed a central black eschar (Fig. 1**e**). Wound swabs demonstrated growth of *P. aeruginosa*, and susceptibility testing revealed unchanged susceptibility patterns. Antibiotic treatment with high-dose piperacillin (4 g qid) was initiated and one week later, aztreonam (2 g bid) was added due to the persistent growth of *P. aeruginosa* in follow-up wound swab cultures, and because the patient had contraindications to aminoglycoside therapy (pre-existing hearing impairment). Recurrent surgical incisions of all the remaining skin lesions were performed. Although the serum IgG and IgM levels were within the normal range, impaired functionality of immunoglobulins (Ig) was assumed due to the underlying CLL [8, 9], and a total of 30 g Ig were administered intravenously once on day 90. Eventually, no bacterial growth was detected in repeat wound swabs and no new skin lesions were observed (Fig. 3) after 23 days of treatment with piperacillin and 15 days of simultaneous treatment with aztreonam. Antibiotic treatment was well tolerated by the patient. The patient was released from the hospital and given ciprofloxacin 750 mg bid for another seven days. Clinical follow-up examination four months after the discontinuation of antibiotic treatment revealed normal inflammation markers and no further skin lesions.

## Discussion and conclusions

In summary, we have reported a case of recurrent EG in a patient with CLL. Due to the patient’s recent travel history, a tropical disease was suspected initially; however, extensive testing for tropical diseases remained negative and wound swabs finally revealed *P. aeruginosa.* It has been reported that EG mainly occurs in immunocompromised, typically neutropenic patients [2]. Interestingly, this patient showed normal and even elevated leukocyte counts with regular neutrophil percentages during the development of the EG lesions, and the serum IgG and IgM levels were also within the normal range. However, we believe that his leukocyte functions and/or humoral immune response might have been impaired due to the treatment with the tyrosine kinase inhibitor ibrutinib and the underlying chronic leukemia. It is well known that CLL increases the risk for a range of infectious diseases, especially with encapsulated bacteria like *Haemophilus influenzae* [10]. Depending on the therapeutic regime and accompanying immunosuppression, further bacterial, fungal and viral infections have been reported [10, 11]. Ibrutinib therapy has been associated with a wide variety of opportunistic infections: invasive fungal diseases (e.g. CNS and pulmonary candidosis and aspergillosis, extrapulmonary *Pneumocystis jirovecii* infection and disseminated *Cryptococcus neoformans* infection), severe invasive bacterial infections caused by a plethora of different pathogens (e.g. *staphylococci, streptococci, mycobacteria, nocardia, listeria*) and, more rarely, viral infections like cytomegalovirus infection. However, to the best of our knowledge, this is the first case report on EG related to ibrutinib treatment. In order to prevent the recurrence of EG, and since the patient’s leukemia was well-controlled, ibrutinib treatment was discontinued.

The primary lesion upon presentation was a livid discoloration on the patient’s left ear, which was caused by a mosquito bite. From this infection site, *P. aeruginosa* presumably disseminated to the trunk and extremities. According to the literature, the most common sites for EG lesions are the perineal region (57%) and lower extremities, whereas only 6% patients develop lesions on the face or trunk [1, 12]. Usually, the primary entry site is difficult to establish due to a multitude of skin lesions.

The prognosis of EG is dependent on multiple factors, including neutropenia and bacteremia, which have been linked to a poor prognosis. Interestingly, this patient never developed diagnostically verifiable *P. aeruginosa* bacteremia, but had lung manifestations in the initial CT scan and a positive BAL culture for *P. aeruginosa*. On his first admission, the patient was treated with antimicrobial drugs for almost six weeks. Previous case reports on EG have described a duration of antibiotic treatment between two to four weeks with favorable clinical outcomes [4, 12, 13]. However, three weeks after the antibiotic treatment was stopped, our patient was re-admitted to the ED with a recurrence of the typical skin lesions, which again revealed the growth of *P. aeruginosa*. This suggests that antibiotic treatment over several weeks was not sufficient to achieve long-term clinical cure in the present case. This might be due to poor penetration of antibiotics into the EG lesions and the untreated underlying immunodeficiency. However, we found no previous descriptions of recurrent EG after successful initial treatment. On the second admission, the patient was treated with high doses of piperacillin and aztreonam (followed by oral treatment with ciprofloxacin), which was combined with consistent surgical incisions of the EG lesions and substitution of Ig. Afterwards, no recurrence of EG was observed. It is possible that a more aggressive surgical debridement of the skin lesions during the first admission might have prevented recurrence of EG – this should be kept in mind in future treatment of EG. The role for Ig treatment in the present case cannot be determined definitely. However, several reports indicate that substitution of Ig improves the outcome of severe infections in CLL patients and humoral immune deficiencies despite normal IgG and IgM serum levels have been described [8, 9, 14, 15].

In conclusion, the present case shows that EG is a rare but serious disease and should be considered in patients with typical skin lesions regardless of a travel history and normal blood leukocyte counts. Despite negative microbial cultures and regression of the skin lesions after almost 6 weeks of antibiotic treatment, we observed a recurrence of EG several weeks after the discontinuation of therapy. Finally, only a long-term multimodal combination therapy consisting of surgical debridement, high-dose combination antimicrobial therapy, and treatment of the impaired immune status led to a permanent clinical cure.

## Data Availability

Data sharing is not applicable to this article as no datasets were generated or analyzed during the current study.

## Abbreviations

EG: Ecthyma gangrenosum
CLL: chronic lymphocytic leukemia
ED: emergency department
BAL: bronchoalveolar lavage
ICU: intensive care unit
CT: computed tomography
CRP: C-reactive protein
Ig: immunoglobuline

## References

[1] Vaiman M, Lazarovitch T, Heller L, Lotan G (2015). Ecthyma gangrenosum and ecthyma-like lesions: review article. Eur J Clin Microbiol Infect Dis.

[2] Martínez-Longoria CA, Rosales-Solis GM, Ocampo-Garza J, Guerrero-González GA, Ocampo-Candiani J (2017). Ecthyma gangrenosum: a report of eight cases. An Bras Dermatol.

[3] Rodriguez JA, Eckardt PA, Lemos-Ramirez JC, Niu J (2019). Ecthyma Gangrenosum of scrotum in a patient with Neutropenic fever: a case report. The Am J Case Rep.

[4] Sluga R, Tersmette M, Sohne M (2019). Hairy cell leukemia presenting with Ecthyma Gangrenosum- a case report. BMC Infect Dis.

[5] Gençer S, Ozer S, Ege Gül A, Doğan M, Ak O (2008). Ecthyma gangrenosum without bacteremia in a previously healthy man: a case report. J Med Case Rep.

[6] Korte AKM, Vos JM (2017). Ecthyma Gangrenosum. N Engl J Med.

[7] Reich HL, Williams Fadeyi D, Naik NS, Honig PJ, Yan AC (2004). Nonpseudomonal ecthyma gangrenosum. J Am Acad Dermatol.

[8] Francis S, Karanth M, Pratt G, Starczynski J, Hooper L, Fegan C, Pepper C, Valcarcel D, Milligan DW, Delgado J. The effect of immunoglobulin VH gene mutation status and other prognostic factors on the incidence of major infections in patients with chronic lymphocytic leukemia. Cancer. 2006;107:1023–33. 10.1002/cncr.22094. 10.1002/cncr.22094 16862572

[9] Freeman JA, Crassini KR, Best OG, Forsyth CJ, Mackinlay NJ, Han P, Stevenson W, Mulligan SP (2013). Immunoglobulin G subclass deficiency and infection risk in 150 patients with chronic lymphocytic leukemia. Leuk Lymphoma.

[10] Nosari A (2012). Infectious complications in chronic lymphocytic leukemia. Mediterr J Hematol Infect Dis.

[11] Morrison VA (2009). Infectious complications in patients with chronic lymphocytic leukemia: pathogenesis, spectrum of infection, and approaches to prophylaxis. Clin Lymphoma Myeloma.

[12] Sarkar S, Patra AK, Mondal M (2016). Ecthyma gangrenosum in the periorbital region in a previously healthy immunocompetent woman without bacteremia. Indian Dermatol Online J.

[13] Kim JS, Ricafort R, Garfein ES, Levin TL (2011). Imaging findings of ecthyma gangrenosum, an unusual complication of pseudomonas sepsis. HSS J.

[14] Grywalska E, Zaborek M, Łyczba J, Hrynkiewicz R, Bębnowska D, et al. Chronic Lymphocytic Leukemia-Induced Humoral Immunosuppression: A Systematic Review. Cells. 2020;9(11). 10.3390/cells9112398 PMC7693361 33147729

[15] Visentin A, Compagno N, Cinetto F, Imbergamo S, Zambello R, Piazza F, Semenzato G, Trentin L, Agostini C (2015). Clinical profile associated with infections in patients with chronic lymphocytic leukemia. Protective role of immunoglobulin replacement therapy. Haematologica.

